# Overcoming immune resistance in hepatocellular carcinoma: insights into mechanisms, predictive factors, and interventional strategies

**DOI:** 10.3389/fimmu.2026.1804366

**Published:** 2026-04-13

**Authors:** Zhenxao Wang, Jun Wu, Qiao Li, Xin Wen

**Affiliations:** 1 Department of Hepatobiliary and Pancreatic Surgery, The Second Hospital of Jilin University, Changchun, Jilin, China; 2 Department of Laboratory Medicine, The Second Hospital of Jilin University, Changchun, Jilin, China

**Keywords:** hepatocellular carcinoma, immune checkpoint inhibitors, immunotherapy resistance, intervention strategies, resistance prediction

## Abstract

Recently, immune checkpoint inhibitors, both as monotherapy and in combination regimens, have become a mainstay of systemic therapies for patients with advanced or unresectable hepatocellular carcinoma (HCC). However, evidence from clinical trials and real-world studies consistently demonstrates that only a subset of patients achieves long-term clinical benefit. A significant proportion of patients exhibit primary resistance or develop acquired resistance during treatment, resulting in low objective response (OR) rates, brief illness control duration, and increased complexity of successive therapeutic lines. The primary constraint in modern practice stems from the lack of reliable, generalizable, and therapeutically applicable techniques for predicting resistance to immunotherapy. This deficiency severely limits effective pretreatment risk stratification, early detection of resistance, and the formulation of rational strategies to overcome immune resistance. Therefore, this review systematically summarizes the primary causes of resistance to HCC immunotherapy, evaluates recent developments in resistance prediction, and focuses particular attention on emerging intervention strategies and potential clinical translation. Thus, it aims to offer practical guidance for enhancing treatment decision-making and to inform the development of future prospective validation studies.

## 1 Introduction

Hepatocellular carcinoma (HCC) is the sixth most common malignant neoplasm globally and the third leading cause of cancer-related mortality; however, its prognosis remains poor, with a 5-year survival rate of 22% (1). Early-stage liver cancer frequently yields a positive prognosis when treated with surgical resection, ablation, liver transplantation, transarterial chemoembolization, and other interventions. A significant number of patients are diagnosed at advanced stages or experience recurrence and metastasis post-treatment, at which point therapeutic options become limited (2–4). The liver’s immune system inherently exhibits a tolerogenic nature due to its continuous exposure to gut-derived antigens and dietary products. These substances are processed by resident cells, including Kupffer cells, liver sinusoidal endothelial cells, and hepatic stellate cells, which promote immune suppression and constrain effector responses. This baseline tolerogenic state is essential for preventing excessive inflammation; however, it also creates an environment that diminishes effective anti-tumor immunity. In HCC, tumor cells exploit these mechanisms by upregulating inhibitory checkpoints, leading to T-cell exhaustion and impaired cytotoxic function. The tumor microenvironment (TME) is a complex ecosystem that includes stromal cells, immune cells, and secreted soluble factors. Stromal cells such as cancer-associated fibroblast (CAFs), endothelial cells, and extracellular matrix components create physical barriers, while immune cells such as regulatory T cells (Tregs), myeloid-derived suppressor cells (MDSCs), and M2-like macrophages play key roles in immune suppression., which collectively suppress effector T and natural killer (NK) cells, thereby facilitating immune evasion (5, 6). Recently, immune checkpoint inhibitors (ICIs), primarily programmed cell death protein 1 (PD-1)/programmed death-ligand 1 (PD-L1) inhibitors and cytotoxic T lymphocyte-associated protein 4 (CTLA-4) inhibitors, have been recognized as the standard first-line treatment for advanced HCC (7). These agents function by reactivating the anti-tumor immune response through the inhibition of interactions between inhibitory receptors on T cells and their respective ligands, thereby counteracting the immune escape mechanisms employed by tumor cells (8–10). In 2022, the U.S. Food and Drug Administration (FDA) approved a combination therapy comprising durvalumab (anti-PD-L1) and tremelimumab (anti-CTLA-4). By 2025, this therapeutic regimen exhibited significant efficacy in the recent HIMALAYA trial, attaining a 5-year overall survival rate of 19.6% in patients with advanced HCC, compared to a 9.4% survival rate observed in the sorafenib cohort (11). Additionally, clinical studies have demonstrated that the combination of ICI with tyrosine kinase inhibitors or anti-vascular endothelial growth factor drugs has markedly improved patient outcomes (12–14). Although ICI therapy has demonstrated significant efficacy in treating HCC, the complex pathogenesis of HCC and the presence of an immunosuppressive TME result in an efficacy rate of less than 20% for ICIs as a monotherapy. The efficacy of combination treatment remains limited, with response rates between 30% and 36% (8, 15, 16).

A common clinical challenge involves patients experiencing tumor progression after initiating ICIs, indicative of primary drug resistance, or within a few months following initial disease control, suggestive of acquired drug resistance. This progression is frequently associated with fluctuations in liver function, an increased risk of immune-related adverse events, and a diminished window for subsequent treatment options (17). Consequently, the primary focus of contemporary research and clinical practice is to develop methodologies for predicting the risk of drug resistance before treatment, recognizing early signs of potential treatment failure, and formulating flexible, personalized treatment strategies tailored to the specific conditions of each patient (16). This review examined immunological resistance mechanisms in HCC, integrated recent clinical research, and assessed drug resistance prediction methods. It highlights strategies to reverse resistance, aiming to enhance personalized immunotherapy for patients with HCC and provide a valuable treatment reference.

## 2 Overview of the mechanisms of immunotherapy resistance in HCC

**Figure 1:**
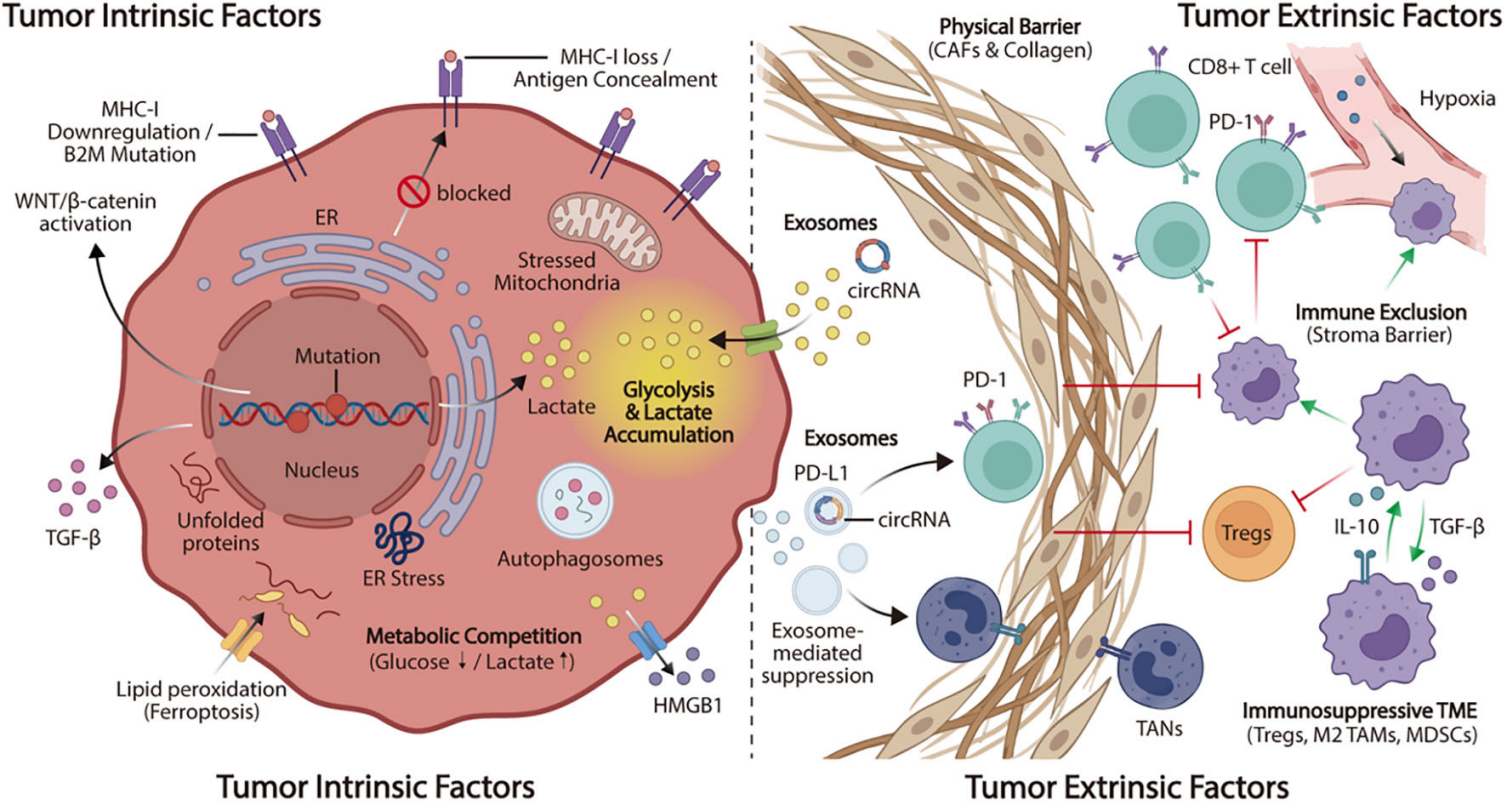
Mechanisms of immunotherapy resistance in HCC. This schematic illustrates the multifaceted nature of resistance to immunotherapy in HCC, divided into tumor-intrinsic and tumor-extrinsic factors. The left panel (Intrinsic Factors) shows mechanisms within the tumor cell, including defects in antigen presentation (MHC-I downregulation, B2M mutation), oncogenic signaling activation (WNT/β-catenin), and secretion of immunosuppressive TGF-β. Metabolic reprogramming leads to lactate accumulation, while stress responses such as ER stress, autophagy, and ferroptosis (releasing HMGB1) contribute to immune evasion. The right panel (Extrinsic Factors) depicts the TME. A dense physical barrier formed by CAFs and collagen prevents T-cell infiltration (immune exclusion). The TME is enriched with immunosuppressive cells, including Tregs, MDSCs, and tumor-associated neutrophils (TANs), which inhibit effector CD8+ T-cells via the PD-1 axis or inhibitory cytokines (IL-10, TGF-β). Additionally, hypoxia and tumor-derived exosomes carrying PD-L1 or circRNA further mediate immunosuppression.

The mechanisms of resistance to ICIs in HCC are complex and can be classified into two distinct categories: Primary drug resistance and acquired drug resistance. Primary drug resistance refers to a tumor that does not respond to immunotherapy from the start of treatment, while acquired drug resistance refers to relapse and disease progression after the initial response (18). Regardless of the resistance category, the fundamental basis is the continuous and dynamic interaction between the intrinsic characteristics of tumor cells, known as tumor-intrinsic factors, and the surrounding microenvironment, termed tumor-extrinsic factors (**Figure 1**) (19).

### 2.1 Tumor-intrinsic factors

The intrinsic factors contributing to immune resistance in HCC primarily include abnormalities in antigen presentation, mutations in genes and signaling pathways, deficiencies in immune surveillance, metabolic reprogramming, autophagy, and ferroptosis mechanisms. First, impaired antigen presentation directly undermines T cells’ ability to recognize antigens, hence diminishing the efficacy of PD-1/PD-L1 blockade therapy in activating and restoring efficacious anti-tumor immune responses (20–23). Studies demonstrated that in HCC, impaired antigen presentation often occurs as downregulation or loss of major histocompatibility complex class I (MHC-I) molecules on tumor cell surfaces, significantly limiting the presentation of tumor-associated antigens or neoantigens to CD8+ cytotoxic T lymphocytes (CTLs). This defect originates from multiple molecular mechanisms, including mutations or epigenetic silencing of key components in the antigen processing machinery, including β2-microglobulin, transporter associated with antigen processing 1, low-molecular-weight protein 2, and low-molecular-weight protein 7 (24–26).

Beyond the intrinsic deficiencies in the antigen presentation machinery, dysregulated oncogenic signaling pathways, specifically Wnt/β-catenin, which is increasingly recognized as pivotal factors that transcriptionally downregulate MHC-I expression and facilitate immune resistance in HCC. Research has demonstrated that abnormal activation of the Wnt/β-catenin signaling pathway can lead to the stabilization and nuclear translocation of β-catenin. This pathway may directly or indirectly inhibit the transcription and expression of MHC-I and related antigen-presenting genes, hence reducing the presentation of novel antigens to CD8+ cytotoxic T lymphocytes CTLs (27). Moreover, dysregulated activation of the Wnt/β-catenin signaling pathway results in a reduced expression of chemokines, including CCL5, impedes dendritic cell recruitment, enhances the presence of inhibitory cells, and stimulates IL-1β production by macrophages. Collectively, these modifications create an immunosuppressive milieu that restricts the infiltration and functionality of CD8+ T cells (28–30). In addition to Wnt/β-catenin, aberrant PI3K/AKT/mTOR signaling also contributes to immune escape in HCC. Hyperactivation of this pathway promotes tumor survival, metabolic rewiring, VEGF production, and the expression of immunosuppressive mediators, thereby impairing effector T-cell infiltration and favoring resistance to ICIs (13). Likewise, disruption of IFN-γ/JAK/STAT signaling weakens antigen processing and presentation, reduces interferon-responsive gene expression, and diminishes tumor susceptibility to T-cell-mediated killing, which has been recognized as a canonical mechanism of immune resistance across tumor types (22).

Furthermore, tumor cells enhance immune resistance by reprogramming metabolism, which amplifies the immunosuppressive effects of signaling pathways and directly establishes a microenvironment that hinders T cell activation through modified metabolic products and nutrient competition. Previous studies demonstrated that HCC cells markedly increase lactic acid production through enhanced glycolysis, known as the Warburg effect (31–33). This metabolic shift leads to the acidification of the TME, which subsequently impairs the cytotoxic functions of CD8^+^ T cells and NK cells. Simultaneously, it facilitates the polarization of M2 tumor-associated macrophages (TAMs) and promotes Treg activity. These processes collectively contribute to primary and acquired resistance to ICIs (32). Recent research has underscored the pivotal role of lipid metabolic reprogramming in immune resistance, extending beyond glucose metabolism. The upregulation of *de novo* lipid synthesis, mediated by (sterol regulatory element-binding protein 1) SREBP1 signaling, not only facilitates tumor proliferation but also induces macrophage polarization and restricts CD8+ T cell infiltration, thereby aiding in immune evasion (34). Furthermore, dysregulated amino acid metabolism, notably characterized by glutamine depletion and tryptophan catabolism through indoleamine 2,3-dioxygenase 1-related pathways, further impairs effector T cell functionality and promotes T cell exhaustion within the TME (35). In addition to the mechanisms mentioned above, epigenetic modifications, including m^6^A RNA methylation and histone modifications, aberrantly activated autophagy pathways, and evasion of ferroptosis, significantly contribute to the development of resistance to immunotherapy in HCC (**Figure 1**) (36–39). These interrelated processes collectively underscore a complex regulatory network that complements alterations in metabolism and signaling pathways, thereby influencing the development of resistance to immunotherapy.

### 2.2 Tumor-extrinsic factors

External factors contributing to drug resistance, especially in the context of acquired resistance, predominantly arise from the TME (40–42). Studies demonstrate that MDSCs and M2-polarized TAMs are significantly abundant in the TME of HCC. These cells inhibit CD8^+^ T cell function and facilitate T cell depletion by secreting inhibitory cytokines, including transforming growth factor-β (TGF-β), interleukin-6 (IL-6), IL-10, and expressing PD-L1 (16, 43–45). Consequently, this leads to resistance against ICIs. In addition to immune-cell-mediated suppression, stromal components also actively participate in resistance. CAFs, together with the extracellular matrix, create a dense physical barrier that restricts the infiltration and antitumor activity of effector immune cells within the TME. CAFs facilitate the formation of a dense interstitial matrix by secreting extracellular matrix (ECM) components, including collagen and fibronectin. This structural barrier obstructs the infiltration of T cells into the central region of the tumor (46, 47). Simultaneously, CAFs can secrete many soluble substances that further inhibit the activity of immunological effector cells. These factors encompass TGF-β and prostaglandin E2 (PGE2), which facilitate the proliferation of regulatory T cells and suppress the proliferation and cytokine secretion of CD8^+^ T cells (48). Recent advancements in high-resolution technologies, including single-cell RNA sequencing (scRNA-seq) and spatial transcriptomics, have significantly clarified the complex heterogeneity of the TME in HCC and its crucial role in facilitating resistance to immunotherapy. Seyhan et al. integrated single-cell and spatial transcriptomic data to substantiate that the heterogeneity within the HCC TME, comprising factors such as epigenetic modifications resulting in CD8^+^ T cell depletion, the development of neutrophil extracellular traps, and dysregulation of the gut microbiome, collectively facilitates immune evasion (49).

Beyond the localized immunosuppressive mechanisms within the TME, tumor cells can exert systemic effects on immune regulation by secreting exosomes. This mechanism further enhances immune evasion and facilitates therapeutic resistance in HCC. Research demonstrates that tumor-derived exosomes transport non-coding RNAs and immunoregulatory proteins, hence modulating immune cells remotely (50). These exosomes circulate through the blood to lymph nodes or bone marrow, enhancing MDSC proliferation and converting TAMs to the M2 phenotype, which increases immune tolerance and drug resistance in HCC (**Figure 1**) (51). Overall, tumor-extrinsic resistance in HCC is not driven by a single cell type or pathway, but by the coordinated action of stromal barriers, suppressive immune populations, and extracellular signaling networks. This complexity also highlights the need to understand the TME as an integrated and evolving system when designing strategies to overcome immunotherapy resistance.

### 2.3 Interactions between tumor-intrinsic and tumor-extrinsic factors

Although tumor-intrinsic and tumor-extrinsic mechanisms are traditionally described as separate categories, increasing evidence indicates that they are tightly interconnected and mutually reinforcing. Genetic alterations in tumor cells can actively reshape the immune microenvironment through secretion of cytokines, chemokines, and extracellular vesicles. For instance, activation of oncogenic pathways such as Wnt/β-catenin or PI3K/AKT can suppress chemokine production required for dendritic cell recruitment, thereby limiting antigen presentation and T-cell priming. Conversely, extrinsic components of the TME, including TAMs, CAFs, and MDSCs, can release soluble mediators that influence tumor cell signaling, metabolic reprogramming, and epigenetic states. This bidirectional interaction establishes a dynamic feedback loop that continuously reinforces immune suppression and promotes resistance to immune checkpoint blockade.

## 3 Prediction of resistance to immunotherapy and assessment of treatment response in HCC

### 3.1 Analysis of blood biomarkers and liquid biopsy techniques

Clinical studies have demonstrated that serum alpha-fetoprotein (AFP), C-reactive protein (CRP), protein induced by vitamin K absence-II (PIVKA-II), IL-6, TGF-β, lactate dehydrogenase (LDH), and various other blood markers correlate with HCC immunotherapy response, reflecting their value in predicting immune resistance (40, 52–57). Researches indicates that following 6 weeks of anti-PD-1 immunotherapy, a reduction in AFP levels exceeding 50% (AUROC, area under the receiver operating characteristic curve: 0.693) or a decrease in PIVKA-II levels by more than 50% (AUROC: 0.672) serves as an indicator of sensitivity to the immunotherapy. This finding offers clinicians a valuable early assessment tool (58, 59). Furthermore, a study of 28 HCC patients treated with PD-1 inhibitors found that those with blood TGF-β levels under 200pg/mL had longer OS and PFS, indicating TGF-β levels may predict immunotherapy response (57). However, multiple meta-analyses and extensive clinical trials related to immunotherapy reveal variations in the application of serum biomarkers (60–63). The predictive value of a solitary biomarker for evaluating drug resistance to ICIs continues to remain contentious, influenced by factors such as varied treatment modalities and patient heterogeneity. Recently, varied scoring methodologies, including neutrophil to lymphocyte ratio (NLR), platelet to lymphocyte ratio (PLR), circulating immune index (CII, white blood cell count (×10 μ/L) divided by lymphocyte ratio (%), the CRP and AFP in immunotherapy (CRAFITY) score, albumin-bilirubin grade (ALBI), TAE, and α-fetoprotein, alkaline phosphatase, and eosinophil (α-FAtE), have exhibited significant advantages in assessing clinical immunotherapy resistance (64–67). These methods combine multiple blood markers with specific patient conditions to improve their evaluative efficacy. CRAFITY assigned 1 point for having an AFP ≥100 ng/ml and 1 point for having a CRP ≥1 mg/dl. Thus, a patient could achieve either 0 (AFP <100 ng/ml and CRP <1 mg/dl), 1 (either AFP ≥100 ng/ml or CRP ≥1 mg/dl), or 2 (AFP ≥100 ng/ml and CRP ≥1 mg/dl) points (68). A cohort analysis of 292 patients who underwent immunotherapy revealed a median OS of 27.6 months for patients classified as CRAFITY low-risk, compared to 6.4 months for those categorized as CRAFITY high-risk. The low-risk group demonstrated a higher disease control rate (68, 69). Furthermore, various research teams have established joint scoring systems that integrate imaging features derived from the CRAFIITY score with clinical indicators and imaging parameters, resulting in a substantial enhancement of predictive accuracy (70, 71).

**Figure 2:**
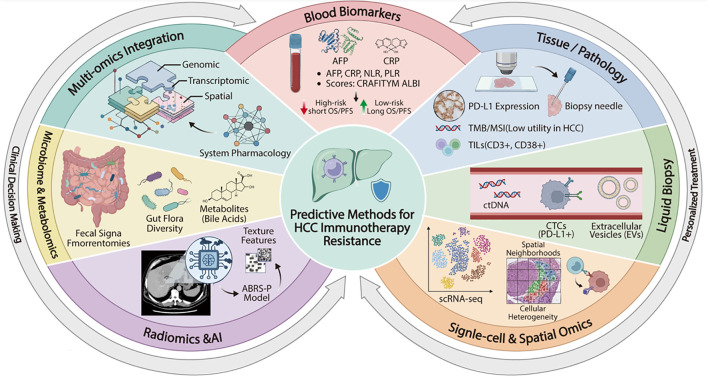
Strategies for predicting immunotherapy resistance in HCC. This diagram illustrates a comprehensive, multi-modal framework used to predict resistance to immunotherapy in HCC patients. The central goal of improving predictive accuracy is supported by seven distinct analytical approaches arranged radially: circulating blood biomarkers, traditional tissue pathology, non-invasive liquid biopsy, advanced single-cell and spatial transcriptomics, AI-driven radiomics from imaging, microbiome and metabolomic profiling, and the integration of multiple omics datasets. By synthesizing data across these diverse biological scales, clinicians can move towards more precise risk stratification, leading to improved clinical decision-making and the development of personalized treatment strategies.

Liquid biopsy is a non-invasive, real-time dynamic monitoring technology that detects multiple biomarkers originating from tumors and host immunity in blood and other body fluids, including circulating tumor DNA (ctDNA), cell-free DNA (cfDNA), circulating tumor cells (CTC), extracellular vesicles/exosomes (EVs), and cell-free RNA (cfRNA). Its value in dynamically guiding HCC immunotherapy, identifying drug resistance, and evaluating prognosis is increasingly acknowledged (72–76). Elena et al. determined that utilizing a threshold of 2.09 ng/μL (AUROC: 0.852), the sensitivity and specificity of ctDNA for predicting HCC resistance to immunotherapy were 81.82% and 87.5%, respectively (77). A prospective cohort study revealed that in a subgroup of 10 patients with HCC undergoing PD-1 therapy, PD-L1-positive circulating tumor cells were identified at baseline in all five patients who exhibited a positive treatment response (78). However, only one of the five patients classified as ineffective responders exhibited PD-L1-positive CTCs, and all ineffective responders experienced disease progression within four months of initiating therapy. These findings highlight the significant role of PD-L1-positive CTCs in evaluating immunoresistance in HCC. Furthermore, ctDNA denotes DNA fragments, typically less than 145 base pairs, derived from necrotic or apoptotic tumor cells (79). An investigation involving 85 patients with HCC undergoing combination immunotherapy utilized gene panels to conduct ultra-deep sequencing of ctDNA mutations across 25 HCC-associated cancer genes. The study revealed that patients with elevated ctDNA levels demonstrated a significantly reduced overall response rate compared to those with lower ctDNA levels and diminished PFS and OS. Beyond ctDNA, total cfDNA burden and cfRNA signatures may also provide complementary information. cfDNA reflects tumor turnover and overall disease dynamics, whereas cfRNA has the potential to capture real-time transcriptional and immune-related changes during treatment. However, their clinical utility in HCC immunotherapy remains investigational and currently lacks standardization (80). Currently, numerous studies focus on exosomes within the extensive research on EVs and immunoresistance in HCC (81–83). Hu et al. reported that exosomes secreted by HCC cells are abundant in circular RNA circCCAR1 (81). Upon uptake by CD8^+^ T cells, circCCAR1 interacts with the WTAP protein within these cells, resulting in CD8^+^ T cell dysfunction and conferring resistance to anti-PD-1 therapy in patients. Most research on EVs remains in the preclinical experimental phase (84). However, advancements in EV separation techniques, improvements in detection technology, and the precise identification of targets highlight their significant potential for clinical application (**Figure 2****).**

### 3.2 Histopathological analysis

The main ICI efficacy biomarkers approved by the FDA include tumor PD-L1 protein levels, tumor mutation burden (TMB), and microsatellite instability (MSI) (85). However, numerous clinical studies have yielded inconsistent findings regarding the applicability of PD-L1 as a predictive marker (85, 86). KEYNOTE-224 and CheckMate040 studies have demonstrated no correlation between baseline tumor PD-L1 expression levels and treatment response. Recent CARES-310 study results indicate that PD-L1 may not be an essential predictive marker (12, 87). However, analyses from the CheckMate459 and GO30140 studies demonstrated a positive correlation between elevated PD-L1 expression levels in tumors and increased immunotherapy response rates (88, 89). This finding indicates that PD-L1 may maintain predictive significance in specific populations. The differences mentioned above may be attributed to variations in detection methodologies, scoring systems, antibody clones, and threshold settings. Alternatively, they may be associated with tumor heterogeneity and dynamic changes in the immunological microenvironment. The predictive significance of TMB or microsatellite instability-high (MSI-H) in HCC is constrained by their low incidence rates (90, 91). The study establishes that the median TMB in HCC was merely 4 mutations per megabase (mut/Mb), with high TMB being infrequent. Moreover, MSI-H was detected in only 0.2% of cases (1 out of 542). Furthermore, a limited number of patients with MSI-H have been reported in several large-scale HCC trials, including IMbrave150, HIMALAYA, and CARES-310. These trials have not established an independent predictive value for MSI-H nor a direct association with drug resistance (80, 92).

Immune resistance in HCC is closely associated with the TME. Research demonstrates that the density of tumor-infiltrating lymphocytes (TILs) is correlated with treatment response (93). A subgroup analysis of the CheckMate 040 study demonstrated that patients who achieved complete response or partial response (PR) exhibited a higher frequency of CD3+ TILs compared to those with stable disease (SD). Accordingly, examining the prevalence of CD3^+^ TILs holds significant value in evaluating immune drug resistance (94). Furthermore, another study confirmed that an elevated proportion of CD38+ cells within tumors correlates with enhanced efficacy of ICIs. This phenomenon may result from enhanced secretion of interferon gamma (IFN-γ) and associated cytokines by macrophages, indicating the potential of CD38^+^ cells as biomarkers for predicting immune resistance (95).

Additionally, scRNA-seq facilitates the investigation of novel cell types, cellular differentiation pathways, biomarkers, and immunological checkpoints, thereby clarifying variations in microenvironments and cellular heterogeneity (96–98). A study utilizing genetic signatures derived from scRNA-seq data effectively identified 21 distinct cell types within the TME before the treatment of advanced HCC (99). The findings revealed that 40% of tumors responding to treatment exhibited pre-existing anti-tumor immune traits, characterized by the infiltration of CD8^+^ effector T cells and pro-inflammatory CXCL10^+^ macrophages. Conversely, primary drug resistance correlated with an enrichment in immunosuppressive myeloid cell populations, including CD14^+^ monocytes and TREM2^+^ macrophages, and the activation of the Notch signaling pathway. One notable advantage of spatial transcriptomics is its ability to maintain information regarding the geographical distribution of tissues. Salié et al. utilized high-dimensional imaging mass spectrometry flow cytometry for spatial single-cell profiling, identifying three unique spatial immunity types—deployed, compartmentalized, and enriched—within a cohort of 101 patients with HCC (100). Notably, the enriched immune architecture was substantially correlated with increased progression-free survival in patients undergoing ICI treatment. Moreover, despite significant tumor heterogeneity, the presence of enriched regions was predictive of long-term survival outcomes. This study highlights the importance of spatial neighborhood analysis in forecasting responses to HCC ICI treatment(**Figure 2**).

### 3.3 Multi-omics and artificial intelligence

Recently, multi-omics integration, including radiomics, microbiomics, metabolomics, genomics, transcriptomics, proteomics, and spatial omics, has emerged as an essential strategy for predicting response in ICIs and elucidating the associated mechanisms in HCC (101–103). A study involving 54 patients with HCC who underwent immunotherapy was conducted to examine the predictive potential of radiomic features extracted from computed tomography (CT) images (104, 105). These features included metrics such as the gray level co-occurrence matrix (GLCM) and the gray level run-length matrix (GLRLM), which were combined with various machine learning models, including deep learning, naive Bayes, and ensemble learning, to create a predictive model. The findings revealed significant differences in specific combinations of traits between patients exhibiting PR and those with SD. Moreover, the model exhibited robust performance in an external validation cohort of 29 patients, accurately predicting short-term treatment efficacy. This study highlights the potential of radiomics as a non-invasive biomarker in the context of HCC treatment. Moreover, the microbiome has recently emerged as a significant focus of research in HCC immunotherapy. Numerous studies have demonstrated that the baseline composition, diversity, and metabolic byproducts of the intestinal microbiota can serve as non-invasive biomarkers for predicting resistance to immunotherapy (106–108). Lee et al. conducted a prospective collection of baseline samples from 94 patients diagnosed with unresectable HCC. Their analysis revealed a significant enrichment of *Lachnoclostridium*, *Lachnospiraceae*, and *Veillonella* in the fecal samples of patients exhibiting an OR to treatment (109). Conversely, an increase in *Prevotella_9* was observed in patients experiencing PD. Furthermore, the bile acids ursodeoxycholic acid and ursocholic acid were identified as abundant in patients with OR and demonstrated a positive correlation with the prevalence of *Lachnoclostridium*. The microbial profile characterized by *Lachnoclostridium* enrichment and *Prevotella_9* depletion serves as an independent predictor of response to HCC immunotherapy.

Recently, the integration of multi-omics analyses has increasingly demonstrated its efficacy in predicting responses to immunotherapy. Li et al. demonstrated that POSTN^+^ extracellular stromal CAFs can affect the immunotherapy response in patients with HCC by integrating single-cell RNA sequencing, spatial transcriptomics, and bulk RNA sequencing technologies (110). Moreover, a recent multi-omics investigation conducted by Li et al. illustrated that the integration of transcriptomic and genomic data with proteomic and metabolomic analyses facilitated the identification of metabolic-immune subtypes predictive of non-responsiveness to PD-1 inhibitors in patients with HCC. Through the incorporation of metabolomic data, the study further substantiated that metabolic reprogramming within the tumor microenvironment fosters immune suppression, which may be mitigated through subtype-specific metabolic interventions (111). The major advantage of multi-omics integration is that it captures resistance across biological scales, from tumor-intrinsic genomics and spatial cell-cell interactions to host-derived microbiome and systemic inflammatory states. However, reproducibility, sample standardization, model interpretability, and external validation remain major barriers to clinical implementation (112).

AI-driven pathology biomarkers offer significant benefit in predicting the response to immunotherapy in HCC. Zeng et al. reported that an AI-based pathology model (ABRS-P) was developed using CLAM deep learning to directly predict the atezolizumab-bevacizumab response signature (ABRS) from digital hematoxylin and eosin (H&E) stained slides of HCC. The findings demonstrated that the median PFS in the ABRS-P high-expression group was significantly greater than that in the low-expression group. Moreover, spatial transcriptomics analysis validated that the expression levels of ABRS and other immune effector genes were upregulated in the high-ABRS-P regions. This study demonstrated that the AI pathological model can function as a cost-effective and rapid biomarker for identifying patients with HCC who are likely to respond positively to atezolizumab-bevacizumab therapy (**Figure 2**) (106).

## 4 Therapeutic strategies and research progress for overcoming immune resistance in HCC

To effectively address immunotherapy resistance, it is crucial to differentiate between primary and acquired resistance mechanisms. Primary resistance is frequently attributed to inadequate immunogenicity or immune rejection of the tumor. Consequently, therapeutic approaches that enhance antigen presentation or facilitate T cell infiltration are deemed appropriate. In contrast, acquired resistance predominantly arises from the tumor’s adaptive evolution under therapeutic pressure, such as through alternative immune checkpoint activation or metabolic adaptation. In such instances, multi-target combination therapies are required to restore therapeutic sensitivity (113). Given the intricate nature of resistance mechanisms, contemporary strategies increasingly emphasize specific biological pathways that drive resistance. These include employing metabolic inhibitors to counteract immunosuppression within the tumor microenvironment, utilizing therapies targeting antigen-presenting pathways to re-establish cytotoxic T cell recognition, and implementing strategies aimed at the tumor microenvironment to diminish immunosuppressive cells and augment immune cell infiltration. This mechanism-oriented framework offers a rational foundation for the development of effective combination therapies (31).

### 4.1 Treatment strategies during the study period

#### 4.1.1 Targeting the immunosuppressive TME

Strategies targeting the TME are designed to “reprogram” this inhibitory milieu into one that fosters immune activity, therefore mitigating primary or acquired drug resistance and enhancing the response rate and durability of immunotherapy. TAM infiltration and M2 polarization are essential components of the TME in HCC. Chen et al. reported that gasdermin E (GSDME) is upregulated in this situation (114). Upon interaction with 3-phosphoinositide-dependent protein kinase-1 (PDPK1), GSDME activates the PI3K/AKT signaling pathway, enhancing M2-like macrophage polarization, suppressing CD8^+^ T cell cytotoxicity, and contributing to resistance against PD-1 antibody therapy. Eliprodil has been identified as a selective inhibitor of GSDME-mediated effects in macrophages. When combined with PD-1 antibody therapy, Eliprodil holds promise as an effective immunotherapeutic strategy. CAFs exhibit a dual function within the TME. Firstly, they facilitate the development of an immunosuppressive TME by orchestrating ECM remodeling, inhibiting immune effector cell activity, and recruiting immunosuppressive cells through the secretion of growth factors and cytokines. This activity contributes to immune evasion, tumor metastasis, and resistance to treatment. Conversely, CAFs can also augment T cell activation via mechanisms associated with the expression of major histocompatibility complex class II molecules, thereby enhancing the effectiveness of immunotherapeutic interventions (115). Xu et al. identified a specific subset of flavin monooxygenase 2 (FMO2)-positive CAFs (116). The presence of FMO2^+^ CAFs enhances the formation of tertiary lymphoid structures and promotes the infiltration of CD8^+^ T cells and M1-like macrophages, thereby increasing the efficacy of anti-PD-1 therapy. Notably, CC motif chemokine ligand 19 (CCL19) plays an essential role in this mechanism. When combined with CCL19, the resistance of HCC to anti-PD-1 therapy can be effectively reduced. Additionally, numerous studies have investigated other immunosuppressive components within the TME, including MDSCs, Tregs, CAFs, vascular endothelial cells, the ECM, and various cytokines and metabolites (49). However, the significant heterogeneity of the TME regarding spatial distribution and its spatiotemporal dynamic evolution under therapeutic pressure poses challenges for current universal treatment strategies to reliably attain efficacy across various patients or even among different lesions within the same patient (**Table 1**).

**Table 1:** Targeting the TME to overcome ICI resistance in HCC.

Drug resistance mechanism	Target	Treatment strategy	Combined immunotherapy approach	Reference
GSDME activates the PI3K-AKT pathway, mediating promotion of macrophage polarization toward an M2-like phenotype	GSDME	Small-molecule Eliprodil selectively inhibits GSDME-mediated effects in macrophages	Anti-PD-1	(114)
CAFs mediate immune resistance	FMO2 + CAF	Recombinant CCL19 protein in combination therapy induces increased infiltration of CD8+ T cells and M1-like macrophages	Anti-PD-1	(116)
CCL2/CCR2 axis induces TAMs toward M2 polarization	SLAMF7	Pharmacological blockade of the CCL2/CCR2 axis combined with upregulation of SLAMF7 expression	Anti-PD-1	(117)
Elevated lactate levels promote M2-type macrophage polarization	SRSF10	Selective inhibitor 1C8 targets the SRSF10/MYB/glycolysis/lactate axis	Anti-PD-1	(118)
M2 polarization and polyamine metabolism in TAMs and MDSCs	MDK	Nanoparticle-delivered therapy (aPD-1 + siRNA@NP) inhibits M2 polarization of TAMs and MDSCs as well as polyamine metabolism	Anti-PD-1	(119)
TAM infiltration	CCL2/CCR2	Abies georgei-derived natural compound 747 antagonizes CCR2, reducing macrophage infiltration	Anti-PD-1	(120)
Trem2 mediates NASH-driven HCC immune resistance	Trem2	Combination with CXCR2 inhibitor AZD5069	Anti-PD-1	(121)
S1P produced by NEK2-highly expressing TAMs promotes resistance	S1P	Use of NEK2 inhibitors or S1P antagonists to target S1P synthesis	Anti-PD-L1	(122)
PLA2G7-highly expressing macrophages exhibit strong immunosuppressive effects	PLA2G7	Darapladib pharmacologically inhibits PLA2G7	Anti-PD-1	(123)
M2 polarization and polyamine metabolism in TAMs and MDSCs	TAM and MDSC	aPD-1@TA-PPPA dual pH-sensitive nanodrug simultaneously inhibits M2 macrophages and MDSCs	Anti-PD-1	(124)
RNasen1 induces TAM polarization via the ALK signaling pathway, leading to immune resistance	RNasen1/ALK	ALK inhibitors target the RNasen1/ALK axis	Anti-PD-1	(125)
Gal1-induced TAMs recruit Tregs via the CCL20-CCR6 axis	Gal1	Inhibition of Gal1-mediated Treg recruitment	Anti-PD-1	(126)
Exosomal circTMEM181 mediates immunosuppressive tumor microenvironment	CD39	Macrophage-specific CD39 knockout	Anti-PD-1	(127)

#### 4.1.2 Targeted metabolic reprogramming

Recent studies have demonstrated that targeting the metabolic reprogramming of tumor and immune cells—including glycolysis/lactate metabolism, lipid metabolism, and amino acid metabolism can effectively reverse the immunosuppressive microenvironment and improve the efficacy of ICIs (128–130). Yasukawa et al. demonstrated that HCC cells lacking ACVR2A may produce and secrete lactic acid by upregulating lactate dehydrogenase A (LDHA) and monocarboxylate transporter 4 (MCT4) (131). This procedure facilitates the accumulation of Tregs, therefore enhancing immunological resistance. Consequently, targeting MCT4 emerges as a novel strategy to alleviate immune resistance in ACVR2A-deficient HCC. Previous research employing diverse genomic analyses, including RNA sequencing, proteomics, and single-cell sequencing, has demonstrated that SREBP1 activation facilitates lipid biosynthesis in tumor cells, induces macrophage reprogramming within the TME toward the M2 phenotype, and diminishes CD8(+) T cell infiltration (34). Notably, CRISPR-mediated knockout of SREBF1 significantly decreases monocyte recruitment and CD206 expression and promotes CD8(+) T cell migration. These findings revealed that inhibiting SREBF1 may counteract immune resistance and enhance the sensitivity to ICIs. Moreover, numerous studies indicate that concurrently targeting LDHA, FASN, or GLS1 with anti-PD-1 therapy can significantly enhance the efficacy of immunotherapy (32). This proposition is founded on mechanisms including lactic acid accumulation, which enhances MDSC and M2 polarization through the GPR81/HIF-1α axis; lipid reprogramming, which triggers ferroptosis in T cells; and glutamine competition, which results in T cell depletion. Amino acid metabolism also contributes to immune resistance in HCC. Competition for glutamine and other key nutrients can impair T-cell activation and effector function, whereas tumor-adaptive metabolic programs can further reinforce suppressive myeloid phenotypes. Although the preclinical evidence is growing, HCC-specific translational data remain less mature than those for lactate and lipid metabolism (132).

#### 4.1.3 Targeted antigen presentation disorder

Antigen presentation defects represent a key mechanism underlying drug resistance in HCC immunotherapy. This phenomenon is primarily characterized by the downregulation or loss of MHC-I molecule expression on tumor cells (133–136). Therefore, tumor antigens are ineffectively presented to CD8^+^ T cells, enabling tumor cells to evade immune recognition and destruction by cytotoxic T lymphocytes. The inhibition of the antigen presentation capability of TAMs can exacerbate the immunosuppressive microenvironment typical of HCC. Research has identified that TAMs expressing the collagen-structured macrophage receptor (MARCO) represent a subset with potent immunosuppressive capabilities (137). MARCO inhibits the release of interferon-beta (IFN-β) by TAMs, reduces the expression of antigen-presenting molecules, and consequently fosters immune resistance. Targeting MARCO-positive TAMs can significantly enhance the efficacy of anti-PD-L1 therapies in HCC. A recent study published in 2026 demonstrated that elemene, a sesquiterpene compound derived from Curcuma species, can significantly enhance the efficacy of anti-PD-1 therapy in HCC. This improvement is accomplished by inhibiting miR-130a-5p, which mitigates the negative regulation of the secreted phosphoprotein (SPP) and MHC-I axis (138). Therefore, this mechanism upregulates MHC-I expression on the surface of tumor cells, restoring antigen presentation and increasing tumor antigen diversity. The restoration of MHC-I expression or the augmentation of antigen presentation by macrophages has become a prominent strategy in recent research. Moreover, strategies including small molecule compounds, targeting peptides, chemokine delivery, and epigenetic regulators have demonstrated efficacy in reversing drug resistance in preclinical models. However, clinical trials are essential to validate their safety and efficacy (**Table 2****).** The expression of antigen-presenting-associated molecules demonstrates considerable variation across different subtypes of HCC and within distinct tumor regions. A study utilizing single-cell RNA sequencing revealed that AFP positive HCC cells exhibited a pronounced upregulation of antigen-presenting genes, including notably elevated expression levels of MHC-I and MHC-II genes, in comparison to AFP negative HCC cells (143). In the future, integrating single-cell sequencing with multi-genomic analysis for biomarker screening is expected to improve the accuracy of therapies aimed at targeting antigen presentation.

**Table 2:** Targeting antigen presentation disorders to overcome ICI resistance in HCC.

Drug resistance mechanism	Target	Treatment strategy	Combined immunotherapy approach	Reference
MARCO + TAM suppress STING-IFN-β signaling pathway activation	MARCO + TAM	Blockade of MARCO + TAM to promote activation of the STING-IFN-I pathway	Anti-PD-1	(137)
BIRC2 inhibits NF-κB p100/p52 processing, thereby suppressing downstream MHC-I expression	BIRC2	BIRC2 antagonist LCL161 blocks BIRC2 function	Anti-PD-1	(139)
Incomplete microwave ablation (IMWA) induces resistance to aPD-1/aCTLA-4 therapy	CTL	smDV-aCTLA-4 stimulates T cells and promotes antigen presentation	Anti-CTLA-4	(140)
Reduced surface expression of antigen/MHC-I complexes on tumor cells	miR-130a-5p/SPP/MHC-I axis	Frankincense (olibanum) enhances surface antigen/MHC-I complex diversity and abundance in HCC via the miR-130a-5p/SPP/MHC-I axis	Anti-PD-1	(138)
CCL3 expression is suppressed in the tumor microenvironment	CCL3-CCR5 pathway	Liver-targeted delivery of CCL3 using recombinant adeno-associated virus (rAAV) enhances macrophage antigen uptake and MHC-II expression via the CCL3-CCR5 axis	Anti-PD-1/Anti-CTLA-4	(141)
BCL9-mediated impairment of antigen presentation function in TAMs	BCL9	BCL9-targeting peptide (hsBCL9Z96) reduces tumor cell BMP4 secretion and CD24 expression	Anti-PD-L1	(142)
PCSK9 promotes lysosomal degradation of MHC-I, leading to defective antigen presentation	PCSK9	Dual-functional RNA regulatory system	Anti-PD-1 antibody	(136)

#### 4.1.4 Targetable signaling pathways

The combination of specific signaling pathways with ICIs presents a novel approach for overcoming drug resistance in HCC immunotherapy. Pathways including Wnt/β-catenin, TGF-β, PI3K/AKT/mTOR, and JAK/STAT are implicated in mediating immune resistance in HCC through various mechanisms (13, 49, 144, 145). Studies demonstrate that abnormal activation of the Wnt/β-catenin signaling pathway can inhibit the expression of CCL5 and NKG2D ligands, reduce the recruitment of dendritic cells (DCs) and NK cells, and consequently result in decreased infiltration of CD8(+) T cells, thereby fostering primary resistance to ICIs (13). The application of Wnt inhibitors, including the Porcupine O-acyltransferase inhibitor CGX1321, counteracts this drug resistance by enhancing T cell infiltration and subsequently reversing the resistance to ICIs. Additionally, Wnt/β-catenin promotes metabolic reprogramming and secretion of immunosuppressive factors by upregulating downstream target genes (c-MYC and cyclin D1), whereas tankyrase inhibitors or niclosamide combined with ICI can effectively overcome immunoresistance (16). In the context of immunoresistance, TGF-β can contribute to T cell depletion and the ICI failure by upregulating the PD-1/PD-L1 axis and suppressing the anti-tumor T cell response. Several studies have demonstrated an enhancement in the immunotherapeutic response in HCC through the application of neutralizing antibodies, including NIS793, or receptor inhibitors (146). The PI3K/AKT/mTOR pathway, alongside Wnt/β-catenin and TGF-β signaling, is crucial in creating an immunosuppressive environment and resistance to ICIs in HCC. Its aberrant activation promotes tumor growth and survival while hindering anti-tumor immunity by reducing antigen presentation and increasing immunosuppressive cell recruitment, such as MDSCs and M2 macrophages. Additionally, mTOR activation affects metabolic reprogramming and impairs effector T cell function, aiding immune evasion (147). The JAK/STAT pathway is crucial in regulating immune response and resistance to immunotherapy in HCC. Disruption of IFN-γ/JAK/STAT signaling can hinder tumor antigen presentation and reduce immune recognition, weakening the response to ICIs. Mutations in JAK1/2 or impaired STAT1 activation can cause resistance by affecting interferon signaling and reducing T cell activity (144, 145).

#### 4.1.5 Targeting T cell immune function

Enhancing cytotoxic immunity through CD8 T cells is an essential approach in addressing drug resistance. Zeng et al. reported that Transmembrane 4 L six family member 1 (TM4SF1) is markedly overexpressed in HCC and functions to inhibit p16/p21-mediated senescence and the activation of the AKT signaling pathway (148). Simultaneously, TM4SF1 upregulates PD-L1 expression and downregulates MHC-I, resulting in the depletion of CD8 T cells. TM4SF1 knockout in an immunocompetent mouse model induces senescence in tumor cells, reverses the immunosuppressive microenvironment, substantially enhances CD8 T cell infiltration and cytotoxic activity, and effectively mitigates resistance to ICIs. Additionally, enhancing the cytotoxic potential of CD8+T cells remains a central goal of immunotherapy. T-cell exhaustion is a major barrier to effective antitumor immunity, and accumulating evidence suggests that combination strategies may help overcome this limitation. In preclinical settings, PD-L1/IL-15 immunocytokine approaches have demonstrated the capacity to strengthen antitumor immune responses and overcome resistance to checkpoint blockade. In HCC, regulatory T cells are increasingly recognized as key mediators of resistance to anti-PD-1 therapy, and disruption of Treg-associated suppressive pathways has been shown to improve the efficacy of PD-1-based treatment (149).

Personalized neoantigen vaccines have demonstrated the ability to elicit strong anti-tumor immune responses across various cancer types. Recent research has developed a vaccine, NeoVAC, composed of seven highly immunogenic neoantigen peptides in combination with clinical-grade polyinosinic-polycytidylic acid (Poly(I: C)) (150). Using orthotopic liver cancer mouse models, alongside techniques including single-cell RNA sequencing, tetramer flow cytometry, and immunofluorescence, the synergistic efficacy of NeoVAC combined with α-PD-1 was assessed, focusing on liver cancer and its associated molecular mechanisms. The findings demonstrate that NeoVAC, when combined with α-PD-1, can enhance the sensitivity of immunotherapy by increasing the infiltration of CD8^+^ tissue-resident memory T cells (TRMs), indicating a promising strategy to overcome immune resistance.

#### 4.1.6 Targeting intestinal flora

Recent studies have demonstrated that the intestinal microbiota influences systemic immune responses, the TME, and drug metabolism through the gut-liver axis, hence significantly contributing to immunotherapy resistance in HCC (107, 151). Zhao et al. reported that *Phocaeicolavulgatus* is common in drug-resistant patients with HCC and suppresses CD8 T cell activation and IFN-γ signaling by reducing indole-3-acetic acid (IAA) synthesis, therefore facilitating resistance against anti-PD-1 therapy (152). In murine models, the genetic knockout of *Phocaeicolavulgatus* or the supplementation with IAA significantly reversed drug resistance and enhanced the density of tumor-infiltrating lymphocytes and the efficacy of ICIs. This study represents the first establishment of a causal association between specific bacterial species and drug resistance in HCC, thereby providing direct evidence for targeted therapeutic interventions. Moreover, research findings demonstrate that responders to ICIs exhibit greater bacterial diversity, with an enrichment of Akkermansia species. Akkermansia supplementation enhances the recruitment of C-X-C motif chemokine receptor 3 (CXCR3)-expressing CD4/CD8 T cells and reduces Tregs, thereby alleviating immune resistance (153). Regulating the intestinal microbiota represents a novel paradigm for addressing drug resistance in immunotherapy for HCC. Specifically, fecal microbiota transplantation (FMT) and targeted bacterial or metabolite therapies exhibit significant promise. Nevertheless, extensive randomized controlled trials are essential to confirm their long-term efficacy and safety.

### 4.2 Treatment strategies during the clinical trial phase

To address the issues of primary and secondary drug resistance in HCC immunotherapy, the paradigm of clinical trials has transitioned from single pathway blockade to adopting a mechanism-oriented, multi-dimensional collaborative strategy (154–157). Current studies focus on the reconfiguration of the TME through the synergistic application of anti-angiogenic agents and localized therapies, alongside the use of novel checkpoint inhibitors, including lymphocyte activation gene-3 (LAG-3) and T-cell immunoglobulin and mucin-domain containing-3 (TIM-3), to combat T cell exhaustion (158, 159). Moreover, efforts are underway to regulate the gut microbiota to enhance the immune response of the ‘gut-liver axis’. Additionally, pioneering investigations are focused on the integration of epigenetic reprogramming, metabolic checkpoint modulation, and the activation of innate immune pathways. These methodologies are designed to recreate the anti-tumor immune cycle, including antigen presentation, effector cell infiltration, and functional restoration, thus providing targeted clinical strategies to counteract immune evasion. This information is corroborated by data from **ClinicalTrials.gov** (**Table 3****).** It is important to acknowledge that the majority of studies related to immunoresistance in HCC, as presented in the table, remain in the preliminary research or exploratory phases. Consequently, comprehensive data on ORR, PFS, and OS are not yet available in the published literature.

**Table 3:** Clinical trial study on ICI resistance in HCC.

Mechanism	Experimental drug	Effect target	Original immunotherapy drug causing resistance	Trial number	Experimental subjects
Gut Microbiota Modulation	FMT + Atezolizumab + Bevacizumab	Gut Microbiota Transplantation + PD-L1 + VEGF	Atezolizumab + Bevacizumab	NCT05750030	Advanced HCC patients with resistance/progression to Atezo+Bev
TME Remodeling + Combined Targeted Therapy	Ivarmacitinib + Camrelizumab + Apatinib	JAK1 + PD-1 + VEGFR2	Prior PD-1/PD-L1 + Targeted Therapy (acquired resistance)	NCT07324473	Unresectable HCC patients with acquired resistance to immune checkpoint inhibitors
TME Remodeling + Combined Targeted Therapy	Regorafenib + Camrelizumab 卤 TUDCA	TKI + PD-1; TUDCA (bile acid derivative, investigated for modulating resistance-related biology)	Prior Anti-PD-1/PD-L1 + Bevacizumab followed by progression	NCT07100392	Advanced HCC; progression after prior PD-1/PD-L1 + bevacizumab combination
Combined Targeted Therapy	Atezolizumab + (Lenvatinib or Sorafenib) vs (Lenvatinib or Sorafenib)	PD-L1 + Multi-Target TKI (VEGFR etc.)	Progression after Atezolizumab + Bevacizumab	NCT04770896	Locally advanced/metastatic HCC; progression after prior atezo+bev systemic therapy
Dual Checkpoint Inhibition	QL1706 (PD-1/CTLA-4 Bispecific Antibody)	PD-1 + CTLA-4	Prior PD-1/PD-L1 Inhibitor (resistant)	NCT06822985	Advanced HCC patients resistant to prior PD-1 immunotherapy
Combined Local Therapy + Dual Checkpoint	QL1706 + Bevacizumab + HAIC/TACE	PD-1/CTLA-4 + VEGF + Local Chemotherapy	Prior Targeted + PD-1/PD-L1 (resistant/intolerant)	NCT07138885	Intermediate-advanced HCC, second-line (targeted-immune resistant)
Alternative Immune Checkpoint + Combined Targeted Therapy	AK104 (Cadonilimab) + Lenvatinib vs Lenvatinib	PD-1/CTLA-4 Bispecific + TKI	Progression after first-line Atezolizumab + Bevacizumab	NCT06984718	Advanced HCC second-line population progressing on first-line atezo+bev
Combined Local Therapy + Targeted	HAIC + Targeted + Immunotherapy (e.g., Camrelizumab + Apatinib)	PD-1 + VEGFR + Local Chemotherapy	Prior TACE + First-Line Targeted-Immuno Failure	NCT05233358	Advanced HCC patients with low response or failure to TACE + first-line targeted-immuno
Combined Local Therapy + Targeted	HAIC + Ramucirumab + PD-1 Inhibitor	VEGFR2 + PD-1 + Local Chemotherapy	Prior Systemic Therapy (second-line)	NCT06441019	Advanced HCC, second-line treatment patients
TME Remodeling + Oncolytic Virus	RP2 (Oncolytic HSV) + Atezolizumab + Bevacizumab	Viral Immune Activation + PD-L1 + VEGF	Atezolizumab + Bevacizumab (second-line)	NCT05733598	Advanced HCC, second-line (after Atezo+Bev)
Targeting Antigen Presentation Defects	Zabadinostat (CXD101) + Geptanolimab	HDAC Inhibitor + PD-1	Progression after prior regimens containing anti-PD-(L)1	NCT05873244	Advanced HCC, immune-related liver cancer (overcoming resistance)
Combined Local Therapy (HAIC)	HAIC (concurrent/sequential) + Targeted + Immuno	Local Chemotherapy + Targeted + Immune	Prior Systemic Therapy	NCT06041477	Advanced HCC (comparing concurrent/sequential)

## 5 Summary

The research and clinical application of immunotherapy for HCC has progressed to a new phase focused on precision medicine. The ongoing advancement of immunotherapy offers novel strategies for advanced HCC treatment; however, drug resistance to immunotherapy limits its clinical application and large-scale development. While AI-based pathology, spatial transcriptomics, and multi-omics integration markedly enhance resistance prediction, several implementation barriers remain. AI models are sensitive to training dataset composition and may exhibit bias or reduced performance in underrepresented HCC subtypes or geographic populations. Spatial transcriptomics requires fresh or optimally preserved tissue and entails high costs, limiting routine clinical deployment. Assay variability, lack of standardized protocols, and the need for large-scale prospective validation across diverse cohorts continue to restrict generalizability. By investigating the mechanisms underlying HCC immunotherapy resistance, conducting comprehensive investigation of immunotherapy diagnosis, and conducting extensive clinical trials, it is expected to significantly enhance the dilemma of HCC immunotherapy resistance, thereby facilitating individualized immunotherapy for patients with advanced HCC, and improving patient survival and prognosis.

## Glossary

ABRS-P: AI-based Radiomics Signature for Prediction (model)
ACVR2A: Activin A Receptor Type 2A
AFP: Alpha-fetoprotein
AI: Artificial Intelligence
ALBI: Albumin-Bilirubin (grade)
ALK: Anaplastic Lymphoma Kinase
B2M: β2-microglobulin
BCL9: B-cell CLL/lymphoma 9
BIRC2: Baculoviral IAP Repeat Containing 2
BMP4: Bone Morphogenetic Protein 4
CAFs: Cancer-associated fibroblasts
CCL19: CC Motif Chemokine Ligand 19
CCL2: CC Motif Chemokine Ligand 2
CCL20: CC Motif Chemokine Ligand 20
CCL3: CC Motif Chemokine Ligand 3
CCL5: CC Motif Chemokine Ligand 5
CCR2: CC Motif Chemokine Receptor 2
CCR5: CC Motif Chemokine Receptor 5
CCR6: CC Motif Chemokine Receptor 6
CD: Cluster of Differentiation (e.g., CD3+, CD38+)
CD24: Cluster of Differentiation 24
CD39: Cluster of Differentiation 39
CD8+: Cluster of differentiation 8 positive
cfDNA: Cell-free DNA
cfRNA: Cell-free RNA
CII: Circulating Immune Index
circCCAR1: Circular RNA CCAR1
circRNA: Circular RNA
circTMEM181: Circular RNA TMEM181
CRP: C-reactive Protein
CRAFITY: CRP and AFP in immunotherapy
CT: Computed Tomography
CTCs: Circulating Tumor Cells
CTLA-4: Cytotoxic T Lymphocyte-associated Protein 4
CTLs: Cytotoxic T Lymphocytes
ctDNA: Circulating Tumor DNA
CXCR3: C-X-C Motif Chemokine Receptor 3
DCs: Dendritic Cells
ECM: Extracellular Matrix
ER: Endoplasmic reticulum
EVs: Extracellular Vesicles
FASN: Fatty Acid Synthase
FDA: U.S. Food and Drug Administration
FMT: Fecal Microbiota Transplantation
FMO2: Flavin Monooxygenase 2
Gal1: Galectin-1
GLCM: Gray Level Co-occurrence Matrix
GLRLM: Gray Level Run-length Matrix
GLS1: Glutaminase 1
GPR81: G Protein-coupled Receptor 81
GSDME: Gasdermin E
HAIC: Hepatic Arterial Infusion Chemotherapy
HCC: Hepatocellular Carcinoma
HDAC: Histone Deacetylase
H&E: Hematoxylin and Eosin
HIF-1α: Hypoxia-inducible Factor 1-alpha
HMGB1: High Mobility Group Box 1
HSV: Herpes Simplex Virus
IAA: Indole-3-acetic Acid
ICIs: Immune Checkpoint Inhibitors
IFN-β: Interferon-beta
IFN-γ: Interferon Gamma
IL-10: Interleukin-10
IL-1β: Interleukin-1β
IL-6: Interleukin-6
IMWA: Incomplete Microwave Ablation
JAK/STAT: Janus Kinase/Signal Transducer and Activator of Transcription
JAK1: Janus Kinase 1
LDH: Lactate Dehydrogenase
LDHA: Lactate Dehydrogenase A
m6A: N6-methyladenosine
mTOR: Mammalian Target of Rapamycin
MARCO: Macrophage Receptor with Collagenous Structure
MCT4: Monocarboxylate Transporter 4
MDK: Midkine
MDSC: Myeloid-derived Suppressor Cell
MDSCs: Myeloid-derived Suppressor Cells
MHC-I: Major Histocompatibility Complex Class I
miR-130a-5p: MicroRNA-130a-5p
MSI: Microsatellite Instability
MSI-H: Microsatellite Instability-high
NEK2: NIMA-related Kinase 2
NF-κB: Nuclear Factor Kappa B
NK: Natural Killer
NKG2D: Natural Killer Group 2D
NLR: Neutrophil-to-Lymphocyte Ratio
OR: Objective Response
OS: Overall Survival
PCSK9: Proprotein Convertase Subtilisin/Kexin Type 9
PD-1: Programmed Cell Death Protein 1
PD-L1: Programmed Death-ligand 1/Programmed Death-Ligand 1
PDPK1: 3-phosphoinositide-dependent Protein Kinase-1
PFS: Progression-Free Survival
PGE2: Prostaglandin E2
PI3K/AKT: Phosphoinositide 3-kinase/Protein Kinase B
PIVKA-II: Protein Induced by Vitamin K Absence-II
PLA2G7: Phospholipase A2 Group VII
PLR: Platelet-to-Lymphocyte Ratio
S1P: Sphingosine-1-phosphate
scRNA-seq: Single-cell RNA Sequencing
SLAMF7: Signaling Lymphocytic Activation Molecule Family Member 7
SPP: Secreted Phosphoprotein
SREBP1: Sterol Regulatory Element-binding Protein 1
SREBF1: Sterol Regulatory Element-binding Transcription Factor 1
SRSF10: Serine/Arginine-rich Splicing Factor 10
TACE: Transarterial Chemoembolization
TAE: (In α-FAtE: α-fetoprotein, Alkaline phosphatase, and Eosinophil)
TAMs: Tumor-associated Macrophages
TANs: Tumor-associated Neutrophils
TGF-β: Transforming Growth Factor-β
TILs: Tumor-Infiltrating Lymphocytes
TKI: Tyrosine Kinase Inhibitor
TMB: Tumor Mutational Burden
TM4SF1: Transmembrane 4 L Six Family Member 1
TME: Tumor Microenvironment
Trem2: Triggering Receptor Expressed on Myeloid Cells 2
Tregs: Regulatory T Cells
TRMs: Tissue-resident Memory T Cells
VEGF: Vascular Endothelial Growth Factor
VEGFR2: Vascular Endothelial Growth Factor Receptor 2
WNT: Wingless-related Integration Site
α-FAtE: α-fetoprotein, Alkaline phosphatase, and Eosinophil.
